# Analysis of heterogeneous dengue transmission in Guangdong in 2014 with multivariate time series model

**DOI:** 10.1038/srep33755

**Published:** 2016-09-26

**Authors:** Qing Cheng, Xin Lu, Joseph T. Wu, Zhong Liu, Jincai Huang

**Affiliations:** 1 Science and Technology on Information Systems Engineering Laboratory, National University of Defense Technology, 410073 Changsha, China; 2 College of Information System and Management, National University of Defense Technology, 410073 Changsha, China; 3 Flowminder Foundation, 17177 Stockholm, Sweden; 4 Department of Public Health Sciences, Karolinska Institutet, 17177 Stock-holm, Sweden; 5 Division of Infectious Disease, Key Laboratory of Surveillance and Early-Warning on Infectious Disease, Chinese Centre for Disease Control and Prevention, Beijing 102206, P. R. China; 6 School of Public Health, Li Kashing Faculty of Medicine, Hong Kong University, Hong Kong Special Administrative Region, China

## Abstract

Guangdong experienced the largest dengue epidemic in recent history. In 2014, the number of dengue cases was the highest in the previous 10 years and comprised more than 90% of all cases. In order to analyze heterogeneous transmission of dengue, a multivariate time series model decomposing dengue risk additively into endemic, autoregressive and spatiotemporal components was used to model dengue transmission. Moreover, random effects were introduced in the model to deal with heterogeneous dengue transmission and incidence levels and power law approach was embedded into the model to account for spatial interaction. There was little spatial variation in the autoregressive component. In contrast, for the endemic component, there was a pronounced heterogeneity between the Pearl River Delta area and the remaining districts. For the spatiotemporal component, there was considerable heterogeneity across districts with highest values in some western and eastern department. The results showed that the patterns driving dengue transmission were found by using clustering analysis. And endemic component contribution seems to be important in the Pearl River Delta area, where the incidence is high (95 per 100,000), while areas with relatively low incidence (4 per 100,000) are highly dependent on spatiotemporal spread and local autoregression.

Dengue fever has spread rapidly within countries and across regions in the past few decades, resulting in an increased frequency of epidemics and severe dengue disease, hyperendemicity of multiple dengue virus serotypes in many tropical countries, and autochthonous transmission in Europe and the USA. Today, dengue is regarded as the most prevalent and rapidly spreading mosquito-borne viral disease among human beings^1^. Prior to 1970, only nine countries experienced dengue epidemics; however, the disease is now endemic in more than 120 countries in Africa, America, the Eastern Mediterranean, Southeast Asia and the Western Pacific^1^. The incidence of dengue has increased 30-fold in the past 50 years, and the geographic range of the virus and its vectors has expanded^2^, with a recent study estimating that there are now 390 million (95% credible interval 284–528) dengue infections per year, of which 96 million (67–136) manifest apparently (any level of disease severity)^3^.

In mainland China, the first outbreak of dengue occurred in Guangdong Province in 1978. Since then, dengue outbreaks have been recorded sequentially in Hainan, Guangxi, Fujian and Zhejiang provinces^4^. From 1990 to 2014, 69,321 cases of dengue including 11 deaths were reported in mainland China, equating to 2.2 cases per one million residents. The highest number was recorded in 2014 (47,056 cases). The number of provinces affected has increased, from a median of three provinces per year (range: 1 to 5 provinces) between 1990 and 2000 to a median of 14.5 provinces per year (range: 5 to 26 provinces) in the period 2001–2014 5. Guangdong province has had the highest incidence of dengue in China (about 94.3% was reported in Guangdong from 2006 to 2014)^6^,^7^. dengue fever is a mosquito-borne viral disease with a strong potential for spatial variation^3^,^8^ and varying transmission^9^. There are a number of reasons why incidences and transmission of dengue vary in time and space^10^. Dengue transmission is highly dependent on environmental factors and human movement. Environmental factors, such as temperature, rainfall and relative humidity, play a significant role in the transmission as well^11^,^12^,^13^,^14^. Limited dispersal distance of the dengue viruses^15^ and daytime biting^16^ imply that human movement should be the primary means by which the viruses spread spatially^17^,^18^. However, human movement and their spatial interaction change over space and time, as individuals vary considerably in the frequency, distance and nature of their movements^9^,^19^. In addition, the heterogeneous incidence levels might be influenced by other unobserved heterogeneity, such as underreporting and cultural differences at geographical scales. To implement efficient control measures, it is crucial to understand the heterogeneous incidence levels and varying transmission of dengue underlying this heterogeneity.

There was a great increase in the incidence of dengue fever in Guangdong Province in 2014. In 2014, the number of dengue fever cases in Guangdong reached a historically high level and exceeded the total number of cases over the previous 10 years. In this study, we perform an analysis of district-level time series of dengue transmission in Guangdong Province in 2014 using a multivariate time series model^20^,^21^, which decomposes dengue risk additively into autoregressive, spatiotemporal and endemic components. The autoregressive and spatiotemporal components represent an autoregression on past counts in the same and in other districts, respectively, and should capture occasional outbreaks and dependencies across regions. The endemic component will describe the background risk of new events by external factors (independent of the history of the epidemic), which in the context of dengue may include seasonality/climate, population, immigration and sociodemographic variables. To account for heterogeneous incidence levels and varying transmission of dengue across districts, region-specific and possibly spatially correlated random effects are introduced in the model^22^,^23^. We identify the potential degree of heterogeneity on a district level through estimated random effect parameters and perform clustering analyses of model fitted value to uncover patterns driving dengue transmission. Understanding the characterization of these patterns might assist in the development of dengue control and prevention strategies in the province.

## Results

**Figure 1:**
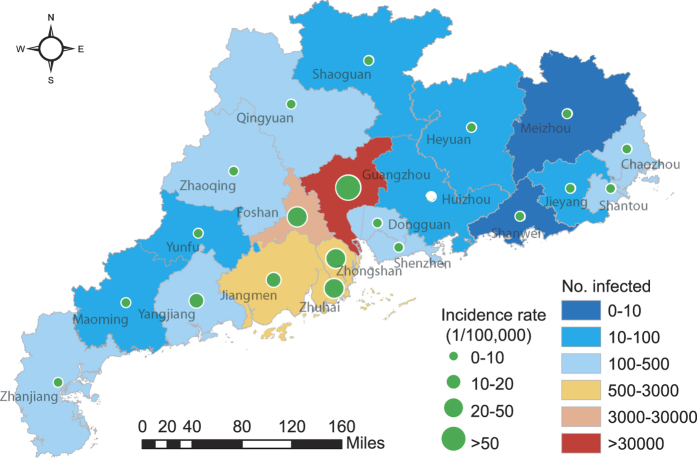
Dengue map at the district level in Guangdong, China, 2014 (created with ArcGis Professional software version 10.2, http://www.esri.com/).

**Figure 2:**
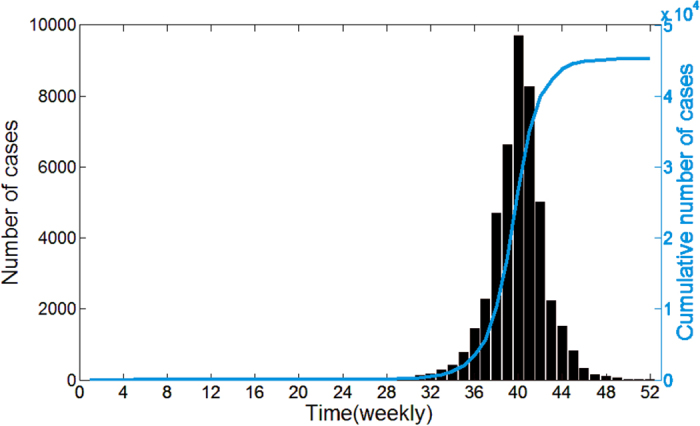
Time series of weekly dengue cases reported, 2014. The blue curve is the cumulative number of dengue cases.

A total of 45,171 dengue cases were reported from 21 districts of Guangdong Province in 2014. The provincial capital, Guangzhou, has 37,394 cases (82.8% of all cases), followed by Foshan (7.8%) and Zhongshan (1.5%) (see Fig. 1). The incidence varies regionally with the highest incidence concentrated in the central districts of Guangdong (see Fig. 1). The majority of cases (42,538, 94.2%) were reported in September and October (weeks 35 to 44). The number of cases peaked in the 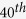 week (9698 cases, about 21.5% of all cases), then decreased towards the end of the year, which indicates a clear seasonal pattern. In addition, the cumulative number of cases grows, similarly to a logistic growth curve (Fig. 2).

In this paper, a multivariate time series model is applied to data on weekly counts of dengue in 21 districts of Guangdong in 2014. In this model, the degree of heterogeneity in real situations is quantified by estimating the random effect value. For model selection in time series models, the comparison of successive one-step-ahead predictions with the actually observed data is used (here, the predictive quality of the models is assessed through one-step-ahead predictions of the last 8 weeks), i.e. fitted value is in turn used as initial value for model update and for calculating all subsequent predictions. In order to capture the effects of significant seasonality, we consider models that differ depending on seasonality parameters (let seasonal term S = 1, 2, 3, details shown in the Method section). Meanwhile, to describe dengue spread in space, we consider two different models (first order neighborhood (Fo) model and power law (PL) model) accounting for spatial interaction between districts. The Fo model assumes that an epidemic can only arrive from directly adjacent districts, and that all districts have the same coefficient for importing cases form neighboring districts. The PL model is considered as a description of spatial interaction as motivated by human travel behavior can be well described by a decreasing power law of the distance or neighborhood order, which assumes the form 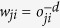, for 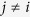 and 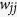 = 0, where 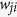 is the weight that describe the strength of transmission form district *j* to district *i*,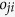 is the order of neighborhood, and 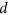 is the decay parameter (the details of both models are described in Method section). According to Table S1 (Supplementary Table S1), all models including PL perform better than models including Fo with respect to all scores. Further improvement of the PL model’s description of human mobility can be achieved by accounting for the district-specific population in the spatiotemporal component, i.e. “PL + pop.” model. Table S1 shows that the “PL + pop.” model with seasonal term S = 3 (denoted as Model D3) outperforms all other models. Therefore, we apply model D3 with random effects to account for district heterogeneity. Parameter estimates for D3 are shown in Table 1.

**Figure 3:**
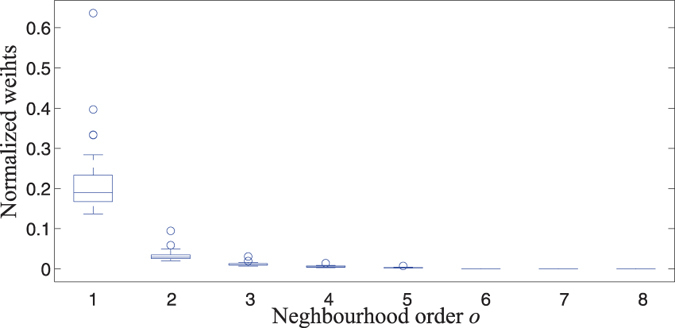
Normalized weights in the multivariate time series model with “PL + pop.” weight.

From the Table 1, the decay parameter estimate is 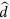 = 2.745 (0.672), which represents a strong decay of spatial interaction for higher-order neighbors because the higher the decay parameter *d*, the less important are higher-order neighbors. Moreover, Fig. 3 shows neighborhood weights *w_ij_* against neighborhood order *o_ij_*, it is obvious that the spatiotemporal component effects mainly account for nearest neighbors dependence.

Note that the heterogeneity of the dengue incidence can thus be captured adequately according to random effect parameter 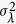, 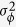, 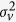 estimated. Little variation in the autoregressive component among districts is found since the variance 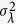 = 0.022 is estimated to be quite small. In contrast, there is considerable spatial variation concerning the endemic coefficient with 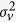 = 8.166 and spatiotemporal coefficient with 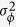 = 3.331. We thus believe that there is significant spatial heterogeneity in the endemic and spatiotemporal component and spatial homogeneity in the autoregressive component.

**Figure 4:**
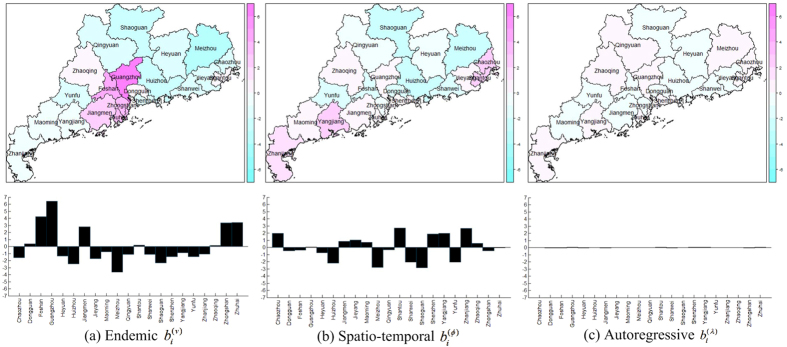
Estimated district-specific random effects in the multivariate time series model. There is considerable variation concerning the endemic coefficient and spatiotemporal coefficient, and there seems to be little variation in the autoregressive coefficient (created with R version 3.3.3, https://www.r-project.org/).

In particular, for the endemic component in Fig. 4(a), there is a pronounced heterogeneity between the Pearl River Delta area (Guangzhou, Foshan, Zhongshan, Jiangmen and Zhuhai, shown in pink) and the remaining districts. Similarly, there is considerable heterogeneity across districts for the spatiotemporal component as shown in Fig. 4(b). However, no clear spatial pattern can be seen. Moreover, all districts exhibit a very low random effect value in the autoregressive component according to the bar chart in Fig. 4(c). There seems to be no significant difference among districts for the random effects in the autoregressive component, thus we infer that the autoregressive effect accounting for dengue transmission in all districts might be homogeneous.

**Figure 5:**
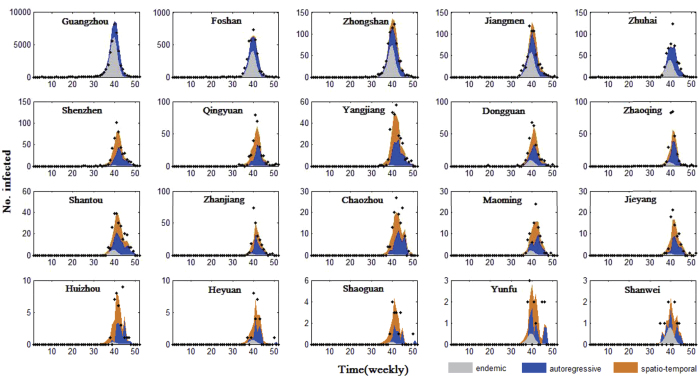
Fitted components in the multivariate time series model for 20 districts with more than 0 cases. Black dots are drawn for weekly counts, the light gray area shows the estimated endemic component, the blue area corresponds to the autoregressive contribution and the orange area corresponds to the spatiotemporal contribution.

For each district, the relative contributions of endemic, autoregressive and spatiotemporal factors in driving the dengue prevalence with time is called the “patterns driving dengue transmission” in this district. An intuitive way of quantifying the relative contributions of the three components is provided by Fig. 5. It shows the fitted component means along with the observed time series for the 20 districts with at least one case. Figure 5 also demonstrates that dengue transmission appears to be synchronous in Guangdong, peaking at the same times of the year in different districts (between weeks 35 and 44). In order to further understand different aspects of dengue transmission patterns and their drivers, we focus on the outbreak period (weeks 35 to 44) and use the Fdp clustering method (see Method Section) to find patterns driving dengue transmission.

**Figure 6:**
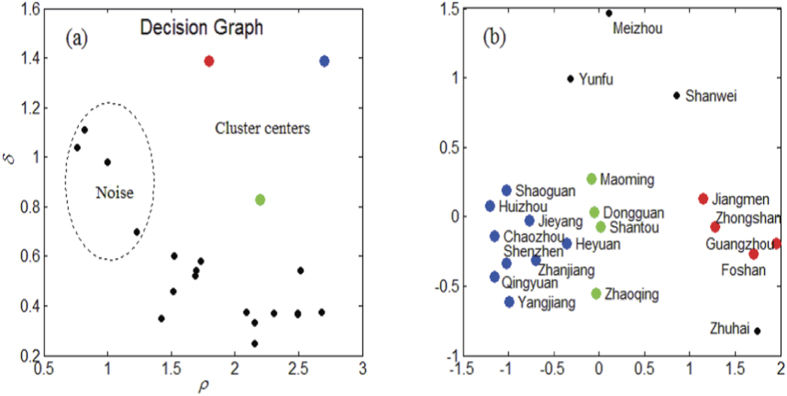
Using Fdp clustering method to find spread patterns. District-specific patterns driving dengue transmission regarded as points: (**a**) Decision graph for the district-specific pattern driving dengue transmission, three cluster centers and four noises are identified; (**b**) Point distribution, different colors correspond to different clusters.

**Figure 7:**
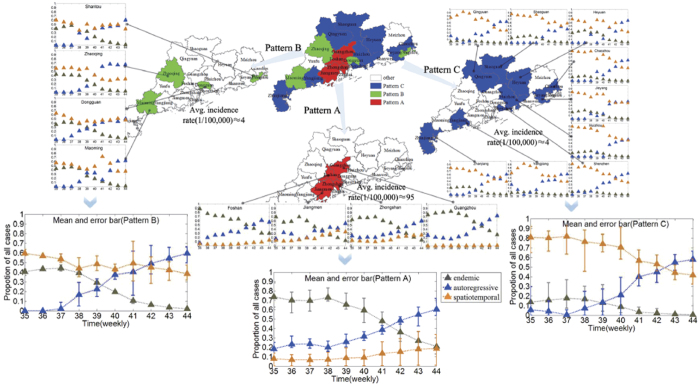
Three patterns and their characteristics. The autoregressive component trends are similar, and their percentages increase in all districts during the dengue outbreak period. In addition, red districts (Jiangmen, Zhongshan, Guangzhou and Foshan, Pattern A) have been mainly affected by the endemic component and are also the ones with the highest overall number of cases. Green districts (Maoming, Dongguan, Zhaoqing and Shantou, Pattern B) are mainly influenced by endemic and spatiotemporal components in the early period, and then the endemic proportion declines gradually, but the spatiotemporal proportion always maintains a relatively high level. The spatiotemporal component of incidence is the most powerful in blue districts (Chaozhou, Shaoguan, Huizhou, Jieyang, Heyuan, Shenzhen, Zhanjiang, Qingyuan and Yangjiang, Pattern C), meaning that a great number of cases are explained via transmission from neighboring districts (created with ArcGis Professional software version 10.2, http://www.esri.com/).

It is obvious that three cluster centers and four noises can be found in the decision graph, as shown in Fig. 6. Thus, we hypothesize that there are three patterns driving dengue transmission in Guangdong, denoted as Patterns A, B, C, respectively (see Fig. 7, red districts belong to Pattern A, green districts belong to Pattern B and blue districts belong to Pattern C). It is worthwhile noting that autoregressive component trends in all districts are similar because there no significant differences among districts for the random effects in the autoregressive component as shown in Fig. 4(c). According to the Fig. 7. It is obvious that the autoregressive’s percentage increase during the dengue outbreak period, which means the local autoregression factor plays an increasingly important role in dengue progress in Guangdong. But the endemic and spatiotemporal components play different roles for different patterns.

Jiangmen, Zhongshan, Guangzhou and Foshan belong to Pattern A (they are in the Pearl River Delta area), have been mainly affected by the endemic component and also have the highest overall number of cases. Especially in the early outbreak period, more than 70% of cases account for endemic component which implies that the socioeconomic, climate and environment might be major factors attribute to dengue outbreak in these districts^24^ because the endemic component describe the background risk of new events by external factors (independent of the history of the epidemic) in our model. In contrast, these districts are estimated to have a relatively low spatiotemporal contribution. Maoming, Dongguan, Zhaoqing and Shantou belong to Pattern B, are mainly influenced by endemic and spatiotemporal components in the early outbreak period, and then the endemic proportion declines gradually, but the spatiotemporal proportion always maintains a relatively high level, i.e. the factors accounting for dengue outbreak in these districts change from endemic and spatiotemporal components to autoregressive and spatiotemporal components. Chaozhou, Shaoguan, Huizhou, Jieyang, Heyuan, Shenzhen, Zhanjiang, Qingyuan and Yangjiang belong to Pattern C. In these districts, the incidence is clearly dominated by the spatiotemporal component, meaning that a great amount of cases is explained via transmission from neighboring districts.

The Fdp clustering method classifies districts independently of the geographic area taking into account only the patterns driving dengue transmission. The majority of cases are clustered in the Pearl River Delta area (with more than 93% of all cases) and the incidence in these districts is relatively high (incidence is about 95 per 100,000). These districts belong to Pattern A and their incidence is clearly dominated by the endemic and autoregressive component, while districts that belong to Patterns B and C with relatively low incidence (incidence is about 4 per 100,000) are highly dependent on spatiotemporal spread and local autoregression.

## Discussion

Our analysis of dengue case report data characterizes considerable heterogenous incidence levels and transmission across districts in the dengue outbreak in Guangdong Province in 2014. The degree of heterogeneity is quantified through random effect parameter estimates; moreover, by using the Fdp clustering method, we explore the characterization of patterns driving dengue transmission, which might be useful for improving our understanding of heterogeneous dengue transmission.

To analyze the spatial and temporal occurrence of dengue and its association with the heterogeneity of environmental characteristics, we fit a multivariate time series model of dengue virus transmission to spatial time series data from Guangdong and compare maximum-likelihood random effect estimates to account for unobserved heterogeneity. To assess the potential for dengue spread in space, both models, the first-order neighbor model and the power law model, accounting for spatial interaction between districts, have been incorporated in the multivariate time series model. We find that the power law model accounting for spatial interaction between districts substantially improves model fit and predictions. We further note that in the best-fitted model D3, there is a strong decay of spatial interaction for higher-order neighbors, which indicates that the spatiotemporal component effects mainly account for nearest neighbor dependence. But this may be limited by the small number of districts in Guangdong: A second-order neighboring district would include all. A better way of accounting for spatial interaction effects would thus be to explicitly incorporate movement network data^25^,^26^. For instance, mobile phone data have been used as proxy for human mobility to achieve improved predictive performance in disease spreading^27^,^28^. But the power law approach to modeling spatial interaction is especially attractive if movement network data are not available^22^.

In this study, we show that the estimated random effect parameters were able to capture the influence of heterogeneity at district level. In particular, there is significant endemic and spatiotemporal variation across districts, while no clear autoregressive heterogeneity is found. Another encouraging finding is the relative importance of the three components in each district and three patterns driving dengue transmission in Guangdong. The Pearl River Delta area in pattern C is highly exhibit a relatively high endemic incidence, which means that a great amount of cases is explained by external factors, such as seasonality/climate, socioeconomic and environment. It seems that the spread of dengue in Pearl River Delta area is more of an endemic than epidemic nature^29^. Our analysis also shows that the districts in Pattern C are highly dependent on the spatiotemporal component and these districts are mostly around the Pearl River Delta area, meaning that a large number of cases in these districts are explained via transmission from neighboring districts, and the Pearl River Delta area is thought to act as a reservoir for the virus from where it can spread to the neighboring districts.

To conclude, this paper underlines the varying transmission of dengue across districts and characteristics of three patterns driving dengue transmission by combing multivariate time series model and clustering method. However, when analyzing spatially stratified time series the assumption of equal transmission rates or incidence levels across all districts is question. For example, dengue transmission might be influenced by factors such as vaccination status in individuals, vector, or environmental factors, Such factors could be incorporated into the multivariate time series model as covariates if they are observable and available. Therefore, it would be necessary to further enrich the model by entering external processes such as mosquito density as covariates in the endemic and epidemic components, and a better way of accounting for spatial interaction would thus be to explicitly incorporate human mobility data.

## Methods

### Study area

Guangdong Province is in Southeast China and has a population of more than 100 million people. It had the highest incidence of dengue in mainland China in 2014, accounting for more than 90% of all cases^5^.Guangdong covers about 180,000 *Km*^2^, with Guangzhou city as the provincial capital; it is one of the most densely urbanized regions in the world and one of the main hubs of China’s economic growth, and it comprises 21 districts (see Fig. 1. In addition, Guangdong is warm and damp all year round with average temperatures ranging from 19 to 26 °C and with a rainy season from April to September^30^.

### Data collection

In this study, we obtained data on dengue fever cases in Guangdong in 2014 from the Chinese Center for Disease Control and Prevention (China CDC). The data were aggregated to weekly counts. In China, all cases of dengue were diagnosed according to the unified diagnostic criteria issued by the Chinese Ministry of Health. Population data for every district in Guangdong in 2014 were retrieved from the Guangdong Statistical Yearbook^31^.

### Multivariate time series model

The multivariate time series model established by Held and Paul^20^,^23^ is designed for spatially and temporally aggregated surveillance data. Let *Y*_*i*,*t*_ denote the number of cases of a specific disease in region *i *= 1, …, *I* at time *t* = 1, …, *T*. The counts are assumed to be negatively binomially distributed with conditional mean


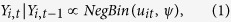


where 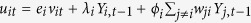 and *Ψ* is an overdispersion parameter such that the conditional variance of *Y*_*i*,*t*_ is *u*_*it*_(1 + *Ψu*_*it*_). e_*i*_*v*_*it*_ is the endemic component and parametrically models seasonal variation and trends. The other two components are observation-driven epidemic components: An autoregressive component *λ*_*i*_*Y*_*i*,*t*−1_ on the number of cases at the previous time point, and a spatiotemporal component 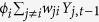 capturing transmission from other units. Each of *v*_*it*_, 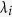, *ϕ*_*i*_ is a log-linear predictor of the form


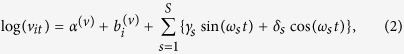



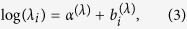



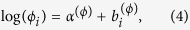


where α^(*v*)^, α^(*λ*)^, α^(*ϕ*)^, are intercepts, *b*_*i*_^(*v*)^, *b*_*i*_^(λ)^, *b*_*i*_^(*ϕ*)^ are regional random effects which account for heterogeneity between districts, and are assumed to follow independently a normal distribution with zero mean and covariance matrix 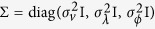, where 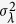, 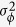, 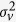, and I is the identity matrix.

The endemic log-linear predictor *v*_*it*_ incorporates a sinusoidal wave of frequency (*ω*_*s*_ are Fourier frequencies, let 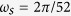 for weekly data in this paper), and *S* is the seasonal parameters. As a basic district-specific measure of disease incidence, the population fraction *e*_*i*_ is included as a multiplicative offset.

The weights *w*_*ji*_ of the spatiotemporal component describe the strength of transmission from region *j* to region *i*, and the neighborhood-based approach to model spatial interaction is especially attractive if movement network data are not available. Thus we consider four different neighborhood-based approaches to measure neighborhood weights.

- The first-order neighborhood model (**Fo**) assumes that an epidemic can only arrive from directly adjacent districts 20, and that all districts have the same coefficient for importing cases from neighboring districts;
- To reflect commuter-driven spread in our model, we scale the district’s susceptibility according to its population fraction by multiplying *ϕ* by 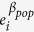 where *e*_*i*_ is the population fraction and 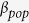 is to be estimated (**Fo + pop**.)^20^;
- To account for long-range case transmission, a power law model (**PL**) is suggested, which assumes the form *w*_*ji*_ = *o*_*ji*_^−*d*^, for *j* *≠* *i* and *w*_*jj*_ = 0, where *o*_*ji*_ is the order of neighborhood, the decay parameter *d* is to be estimated^32^;
- Based on the power law model used to measure neighborhood weights, we scale the district’s susceptibility according to its population fraction by multiplying *ϕ* by 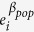 where *e*_*i*_ is the population fraction and *β*_*pop*_ is to be estimated (**PL + pop**.).

The estimation of parameters involves integration of the likelihood with respect to the random effects which cannot be obtained analytically. Paul and Held^23^ suggest a penalized likelihood approach for inference, where variance components are treated as known when estimating the fixed and random effects. The variance components themselves are estimated through maximizing the approximated marginal likelihood obtained via a Laplace approximation. However, classical model choice criteria such as Akaike’s Information Criterion (AIC) cannot be used straightforwardly for models with random effects. Therefore, the performance of the power law models and the first-order formulations is compared by one-step-ahead forecasts assessed with strictly proper scoring rules: the logarithmic score (logS) and the ranked probability score (RPS)^33^, and lower scores correspond to better predictions.

### Clustering method

Based on the multivariate time series model, for each district, endemic, autoregressive and spatiotemporal components are different over time. For each district, the relative contributions of endemic, autoregressive and spatiotemporal factors in driving the dengue prevalence with time are called the “pattern driving dengue transmission”. Let *end*_*i*,*t*_, *ar*_*i*,*t*_ and *ne*_*i*,*t*_ denote endemic, autoregressive and spatiotemporal components accounting for the proportion of number of cases in district *i* = 1, …, *I* at time *t* = 1, …, *T* respectively, i.e. *end*_*i*,*t*_ = *e*_*i*_*v*_*it*_/*Y*_*i*,*t*_, *ar*_*i*,*t*_ _*=*_ *λ*_*i*_*Y*_*i*,*t*−1_/*Y*_*i*,*t*_ and 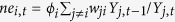. Then the pattern driving dengue transmission of district *i* from *t* = *s* to *t* = *e* can be denoted as 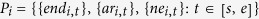. In fact, the pattern driving dengue transmission is a multivariate time series.

First, we define the distance between two patterns driving dengue transmission by using the Euclidean distance


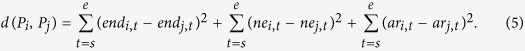


Then, we revise the Fdp clustering method proposed by Alex Rodriguez *et al*.^34^ as below: The pattern driving dengue transmission in each district is regarded as a data point. The local density *ρ*_*i*_ of data point *i* is defined as


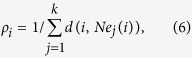


where *d*(*i*, *j*) represents the distance between data point *i* and *j*, and *Ne*_j_(*i*) represents the *j*^th^-nearest neighbor of data point *i*. In this paper, let *k* = 3.

The distance *δ*_*i*_ of data point *i* is measured by computing the minimum distance between the data point *i* and any other data point with higher density^34^:


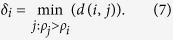


If the data point is with the highest density, we conventionally take 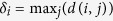. Note that *δ*_*i*_ is much larger than the typical nearest neighbor distance only for points that are local or global maxima in the density. Thus, cluster centers are recognized as points for which the value of *δ*_*i*_ is anomalously large. After the cluster centers have been found, each remaining point is assigned to the same cluster as its nearest neighbor of higher density. Some points have a relatively high *δ* and a low *ρ* because they are isolated; they can be considered clusters composed of a single point, namely noise.

## Additional Information

**How to cite this article**: Cheng, Q. *et al*. Analysis of heterogeneous dengue transmission in Guangdong in 2014 with multivariate time series model. *Sci. Rep.* **6**, 33755; doi: 10.1038/srep33755 (2016).

## Supplementary Material

Supplementary Information

## Figures and Tables

**Table 1:** Estimated model parameters (with standard errors).

Model	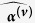 (se)	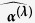 (se)	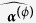 (se)	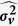	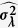	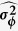	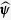 (se)	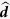 (se)
D3 (“PL + pop.”, S = 3)	−2.934 (0.811)	−0.899 (0.101)	1.769 (3.324)	8.166	0.022	3.331	0.130 (0.022)	2.745 (0.672)
