# Supplementary material for: Analysis of heterogeneous dengue transmission in Guangdong in 2014 with multivariate time series model
Source: Sci Rep. 2016 Sep 26;6:33755. doi: 10.1038/srep33755 (PMC5036033; doi:10.1038/srep33755)
Supplement: Supplementary Information [file srep33755-s1.pdf]

SUPPLEMENTARY INFORMATION

# Analysis of heterogeneous dengue transmission in Guangdong in 2014 with multivariate time series model

Qing Cheng[1,2,*], Xin Lu[2,3,4,5], Joseph T. Wu [6], Zhong Liu[1,2] , Jincai Huang[1,2]

[1] Science and Technology on Information Systems Engineering Laboratory, National University of Defense Technology, 410073 Changsha, China

[2] College of Information System and Management, National University of Defense Technology, 410073 Changsha, China

[3] Flowminder Foundation, 17177 Stockholm, Sweden

[4] Department of Public Health Sciences, Karolinska Institutet, 17177 Stockholm, Sweden

[5] Division of Infectious Disease, Key Laboratory of Surveillance and Early-Warning on Infectious Disease, Chinese Centre for Disease Control and Prevention, Beijing 102206, P. R. China

[6] School of Public Health, Li Kashing Faculty of Medicine, Hong Kong University, Hong Kong Special Administrative Region, China

*Corresponding author: sgggps@163.com

Table S1. Shows the mean scores based on one-step-ahead predictions. Model D3 is compared with the remaining models; the Monte Carlo p-values are based on permutation tests for paired observations (9999 permutations). Bold indicates the best model, i.e. the model with the lowest score

| Model | S | $\overline{\boldsymbol{logS}}$ | (p-value) | $\overline{\boldsymbol{RPS}}$ | (p-value) |
|---|---|---|---|---|---|
| With Fo weight model | | | | | |
| A1 | 1 | 1.638 | (0.0796) | 5.542 | (0.0071) |
| A2 | 2 | 1.611 | (0.0766) | 6.158 | (0.0081) |
| A3 | 3 | 1.595 | (0.0573) | 5.232 | (0.0076) |
| With Fo+pop. weight model | | | | | |
| B1 | 1 | 1.650 | (0.0801) | 5.748 | (0.0074) |
| B2 | 2 | 1.610 | (0.0909) | 6.168 | (0.0085) |
| B3 | 3 | 1.589 | (0.0599) | 5.197 | (0.0076) |
| With PL weight model | | | | | |
| C1 | 1 | 1.599 | (0.1451) | 5.644 | (0.0091) |
| C2 | 2 | 1.569 | (0.0215) | 5.226 | (0.0076) |
| C3 | 3 | 1.524 | (0.1066) | 1.509 | (0.7993) |
| With PL+pop. weight model | | | | | |
| D1 | 1 | 1.599 | (0.1424) | 5.665 | (0.007) |
| D2 | 2 | 1.574 | (0.0154) | 5.347 | (0.0081) |
| D3 | 3 | 1.520 | | 1.506 | |
